# Dynamic HALP Score as a Time-Dependent Prognostic Biomarker in Multiple Myeloma Patients Undergoing Autologous Stem Cell Transplantation

**DOI:** 10.3390/cancers18101570

**Published:** 2026-05-12

**Authors:** Öznur Aydın, Onur Şahin, Enis Akca, Derya Deniz Kürekci, Sude Hatun Aktimur, Engin Kelkitli, Mehmet Turgut

**Affiliations:** 1 Department of Hematology, Faculty of Medicine, Samsun University, Samsun 55080, Türkiye; derya.kurekci@samsun.edu.tr (D.D.K.); shatun.aktimur@samsun.edu.tr (S.H.A.); 2 Department of Hematology, Faculty of Medicine, Ondokuz Mayıs University, Samsun 55139, Türkiye; onur.sahin@omu.edu.tr (O.Ş.); engink@omu.edu.tr (E.K.); turgutm@omu.edu.tr (M.T.); 3 Department of Internal Medicine, Samsun City Hospital, Samsun 55080, Türkiye; enis.akca@saglik.gov.tr

**Keywords:** multiple myeloma, autologous stem cell transplantation, HALP score, progression-free survival, prognostic biomarker, immunonutritional status, maintenance therapy

## Abstract

Background and Objectives: The hemoglobin, albumin, lymphocyte, and platelet (HALP) score is an immunonutritional biomarker reflecting systemic inflammation, nutritional status, and immune competence. Its dynamic behavior and prognostic relevance in multiple myeloma (MM) following autologous stem cell transplantation (ASCT) remain insufficiently characterized. Materials and Methods: In this retrospective cohort study, 95 MM patients undergoing ASCT were analyzed. HALP scores were calculated at diagnosis and at post-transplant day +100, and dynamic changes (ΔHALP) were assessed. Associations with overall survival (OS) and progression-free survival (PFS) were evaluated using Kaplan–Meier and multivariable Cox regression analyses, with maintenance therapy incorporated as a covariate. Results: During a median follow-up of 50 months (range: 11.0–144.0), 36.8% of patients progressed or relapsed, and 23.2% died. Baseline HALP was associated with both OS and PFS in unadjusted analyses; however, the inverse association at diagnosis was substantially attenuated after adjustment for maintenance therapy (HR: 0.71, 95% CI: 0.28–1.80; *p* = 0.466). HALP at day +100 showed a robust association with PFS that strengthened after adjustment (HR: 3.27, 95% CI: 1.45–7.38; *p* = 0.004). The discriminative performance for treatment response was limited, with 95% CIs encompassing AUC = 0.50. Conclusions: Post-transplant HALP at day +100 emerged as the most robust HALP-based prognostic indicator for PFS. Given the small sample size, limited OS events (*n* = 22), use of outcome-driven cut-offs, and absence of cytogenetic and minimal residual disease data, dynamic HALP assessment may provide exploratory prognostic information warranting validation in larger, prospective, multi-center cohorts.

## Simple Summary

The hemoglobin, albumin, lymphocyte, and platelet (HALP) score is a simple, accessible composite index reflecting nutritional and immune status. This study evaluated whether HALP—measured at diagnosis, at post-transplant day +100, and as a dynamic change (ΔHALP)—associates with survival outcomes in 95 multiple myeloma patients undergoing autologous stem cell transplantation. Post-transplant HALP at day +100 emerged as the most robust prognostic indicator for progression-free survival, with its association persisting and strengthening after adjustment for maintenance therapy. In contrast, baseline HALP associations were substantially attenuated after adjustment, and HALP could not predict treatment response. Given the exploratory nature of these findings, external validation in larger, prospective cohorts is required before any clinical application can be considered.

## 1. Introduction

Multiple myeloma (MM) is a hematologic neoplasm characterized by clonal proliferation of plasma cells and accounts for approximately 2% of all malignancies worldwide [1]. According to global cancer registry data, 176,404 new cases of MM were diagnosed in 2020, representing nearly 14% of all leukemia, lymphoma, and myeloma cases combined [2]. Incidence rates have increased over recent decades, particularly in developed countries [3]. MM predominantly affects older individuals, with a median age at diagnosis of 69 years, and despite substantial advances in therapy, it remains an incurable disease [1,2].

For transplant-eligible patients with newly diagnosed MM, autologous stem cell transplantation (ASCT) following induction therapy constitutes the standard of care [4,5]. The incorporation of monoclonal antibodies such as daratumumab into induction and maintenance regimens has led to deeper responses and improved survival outcomes [6,7]. Nevertheless, considerable heterogeneity in clinical course persists even among patients with comparable disease stage and treatment exposure. The International Staging System (ISS) and the Revised ISS (R-ISS), based on biochemical and cytogenetic parameters, are widely used in routine clinical practice [8]. However, these systems do not fully capture host-related factors such as nutritional and inflammatory status, underscoring the need for simple and accessible complementary biomarkers.

The hemoglobin, albumin, lymphocyte, and platelet (HALP) score was first introduced by Chen et al. as a prognostic indicator in gastric cancer [9]. Subsequent studies have evaluated HALP across various malignancies, including gastrointestinal, genitourinary, lung, and breast cancers, generally demonstrating an association between low HALP values and adverse outcomes [10,11,12,13,14,15]. The HALP score integrates parameters reflecting nutritional status, systemic inflammation, and immune competence into a single composite index. Platelet count, lymphocytes, hemoglobin, and albumin reflect different aspects of tumor biology and host response. Platelets have been associated with tumor progression and adverse outcomes, whereas lymphocytes play a central role in antitumor immunity. Hemoglobin and albumin are well-established indicators of general condition and nutritional status. Therefore, the HALP score may provide a comprehensive measure of the interplay between inflammation, immunity, and nutritional status. It is calculated as hemoglobin (g/dL) × albumin (g/dL) × lymphocyte count (µL)/platelet count (µL), and low HALP values have been associated with poor overall survival and inferior treatment response in multiple tumor types [16]. However, the direction and consistency of these associations may vary across different disease contexts.

Beyond solid tumors, the prognostic value of HALP has also been explored in hematologic malignancies. In diffuse large B-cell lymphoma (DLBCL), low HALP scores have been independently associated with inferior survival outcomes [17,18].

In multiple myeloma specifically, the prognostic role of HALP has been evaluated in a limited number of studies. Solmaz et al. identified a cutoff value of 28.8 using receiver operating characteristic (ROC) analysis and reported longer overall survival in patients with higher HALP scores [19]. Similarly, Zhang et al. determined a cutoff value of 41 using the X-tile method and demonstrated that low HALP was associated with survival outcomes alongside R-ISS stage [20]. However, these studies have primarily focused on baseline HALP values at diagnosis, and data regarding post-transplant HALP dynamics—particularly HALP at day +100 after ASCT and treatment-related changes (ΔHALP)—remain limited.

Therefore, the aim of the present study was to evaluate the associations of baseline HALP score, day +100 post-ASCT HALP score, and ΔHALP with overall survival (OS) and progression-free survival (PFS) in patients with MM undergoing ASCT. In addition, we assessed their relationship with treatment response at day +100 post-transplantation.

## 2. Materials and Methods

### 2.1. Study Design and Population

This retrospective cohort study included patients with MM who underwent ASCT at the Department of Hematology, Ondokuz Mayıs University Faculty of Medicine, between October 2019 and December 2022.

The study protocol was approved by the Samsun University Non-Interventional Clinical Research Ethics Committee (Protocol code: GOKAEK 2025/10/4). The requirement for informed consent was waived due to the retrospective design. The study was conducted in accordance with the Declaration of Helsinki.

### 2.2. Inclusion and Exclusion Criteria

Eligible patients had:

(1) A histopathologically confirmed diagnosis of MM;
(2) Undergone ASCT during the study period;
(3) Available day +100 post-transplant evaluation;
(4) Complete laboratory data required for HALP calculation at diagnosis and day +100.

Patients who underwent tandem or allogeneic transplantation, died before day +100, or were younger than 18 years were excluded.

### 2.3. Data Collection and Variables

Data were obtained from electronic medical records and patient files. Collected variables included age, sex, myeloma subtype, ISS stage, induction regimens, maintenance therapy (received vs. not received, regimen, and duration), laboratory parameters (hemoglobin, albumin, lymphocyte count, platelet count), and survival outcomes.

Three HALP-related variables were analyzed: baseline HALP at diagnosis, HALP at day +100 post-ASCT, and ΔHALP (day +100 minus baseline).

ISS staging was defined according to standard beta-2 microglobulin and albumin thresholds [8].

### 2.4. Outcomes

The primary endpoints were OS and PFS. OS was defined as the time from diagnosis to death from any cause. PFS was defined as the time from diagnosis to progression or death. Patients without events were censored at the last follow-up.

Treatment response at day +100 was evaluated according to the International Myeloma Working Group (IMWG) response criteria [21] and categorized as good response [Stringent complete response (sCR) + very good partial response (VGPR)] versus suboptimal response [partial response (PR) + stable disease (SD) + progressive disease (PD)]. Following clarification with the treating clinician, all patients initially recorded as complete response (CR) fulfilled IMWG criteria for sCR and were therefore classified as sCR.

### 2.5. Sample Size Considerations

All consecutive eligible patients were included. No formal a priori sample size calculation was performed due to the retrospective design.

The events-per-variable (EPV) rule was considered to evaluate the stability of multivariable models. In the maintenance-adjusted multivariable models, 21 deaths were observed for OS (EPV = 4.2 with five covariates) and 39 events for PFS (EPV = 7.8). Conventional EPV recommendations were not met for OS models; therefore, OS findings were interpreted as exploratory and hypothesis-generating rather than definitive [22,23].

Post hoc power analysis was not performed. Because post hoc power calculations largely reflect observed *p*-values, this approach was not applied. Instead, effect sizes were reported with 95% confidence intervals.

### 2.6. Statistical Analysis

Continuous variables were summarized as mean ± standard deviation or median [range], as appropriate. Categorical variables were presented as frequencies and percentages.

Group comparisons were performed using the Mann–Whitney U test or Kruskal–Wallis test with Dunn’s post hoc correction. Correlations were assessed using Spearman’s coefficient.

Optimal HALP cut-off values were determined using the X-tile algorithm. Given the outcome-driven nature of this approach, these analyses were interpreted in an exploratory context.

Sensitivity analyses compared three HALP cut-offs: a literature-based value (28.8) previously reported in a Turkish multiple myeloma cohort [19], the cohort median (32.0), and the X-tile-derived value (58.87).

Survival curves were estimated using the Kaplan–Meier method and compared using log-rank tests. Associations with OS and PFS were evaluated using univariate and multivariable Cox proportional hazards models, with results reported as hazard ratios (HRs) and 95% confidence intervals. The proportional hazards assumption was verified using Schoenfeld residuals.

Multivariable Cox models included the HALP variable, age, sex, ISS stage (III vs. I–II), and maintenance therapy (received vs. not received). The analysis was restricted to patients with known maintenance therapy status (*n* = 90); five patients with unknown maintenance status were excluded from these models.

To address collinearity between HALP and serum albumin (a constituent of the HALP composite), HALP and albumin were not entered into the same model. Two alternative multivariable specifications were prespecified to address collinearity between HALP and serum albumin: Model A (HALP-based) included the HALP variable, age, sex, ISS stage, and maintenance therapy, whereas Model B (albumin-based) replaced HALP with serum albumin.

Restricted cubic spline analyses were performed to explore potential non-linearity.

For the response prediction analysis, ROC curves were plotted and the area under the curve (AUC) with 95% confidence intervals was estimated using non-parametric bootstrap resampling (1000 replications).

To assess the incremental prognostic contribution of dynamic HALP parameters (HALP at day +100 and ΔHALP) over baseline HALP, nested Cox proportional hazards models were compared using the likelihood ratio test (LRT). The base model included baseline HALP (low vs. high), age, sex, and ISS stage; each dynamic HALP parameter was added separately. Model fit was further evaluated using the change in the Akaike Information Criterion (ΔAIC).

All analyses were performed using Python (version 3.12; lifelines and statsmodels libraries), Jamovi version 2.6.44, and JASP version 0.95.4.0. A two-sided *p*-value ≤ 0.05 was considered statistically significant.

## 3. Results

A total of 95 patients with MM who underwent ASCT were included in the study. The median age of the cohort was 65.0 years (range: 42.0–75.0), and 60.0% of the patients were male. According to ISS stages, more than half of the patients (52.6%) were classified as Stage I, while 27.4% were Stage II and 20.0% were Stage III.

The median time from diagnosis to transplantation was 7.3 months (range: 2.0–75.0). Two patients (2.1%) received daratumumab-based induction regimens (anti-CD38 monoclonal antibody) as part of their pre-transplant therapy.

At the day +100 post-transplant evaluation, sCR was achieved in 26.3% of patients and VGPR in 56.8%, resulting in an overall good response rate (sCR + VGPR) of 83.1%. The median follow-up duration was 50.0 months (range: 11.0–144.0).

Maintenance therapy was administered to 75 of 95 patients (78.9%); 15 patients (15.8%) did not receive maintenance, and maintenance status was unknown for 5 patients (5.3%). Among recipients, lenalidomide monotherapy was the most common regimen (*n* = 56, 74.7%), followed by combination regimens (*n* = 11, 14.7%) and other agents (*n* = 8, 10.7%). Median maintenance duration was 41 months (range: 14–140).

During follow-up, disease progression or relapse occurred in 36.8% of patients, and death was observed in 23.2% (Table 1).

The median HALP score increased from 32.03 at diagnosis (range: 3.48–468.74) to 57.83 at day +100 post-transplantation (range: 8.58–831.66). The median ΔHALP value was 19.47 (range: −389.77 to 804.81) (Table 2).

The baseline HALP score at diagnosis was significantly higher in males compared to females (median: 37.87 vs. 26.13; *p* = 0.011). ΔHALP differed significantly across ISS stages (*p* = 0.043), with post hoc analysis revealing a difference between Stage I and Stage II. No significant differences in HALP parameters were observed according to day +100 response, progression, or survival status (all *p* > 0.05; Table S1). Correlation analysis between HALP scores and age demonstrated that none of the three HALP variables were significantly correlated with age (HALP at diagnosis: *r* = −0.001, *p* = 0.994; HALP at day +100: *r* = 0.120, *p* = 0.248; ΔHALP: *r* = 0.063, *p* = 0.543) (Table S2).

For PFS, statistically significant log-rank results were observed for cut-offs based on HALP at diagnosis, HALP at day +100, and ΔHALP (*p* = 0.035, *p* = 0.025, and *p* = 0.018, respectively). In contrast, for OS, no HALP-based cut-off demonstrated a statistically robust association, with only a borderline non-significant result observed for HALP at diagnosis (*p* = 0.083), while the remaining HALP-based cut-offs were not significant (Table 3).

The performance of HALP scores in predicting day +100 treatment response was evaluated using ROC analysis. None of the three HALP variables demonstrated significant discriminative ability; bootstrap-derived 95% confidence intervals for the AUC values encompassed 0.50, indicating that the observed discrimination was not statistically distinguishable from chance (Tables S3 and S12, Figure S4).

In Kaplan–Meier analysis, no statistically significant separation in OS was observed between groups stratified by HALP score at diagnosis, HALP score at day +100, or ΔHALP (all *p* > 0.05) (Table 4 and Table S5, Figures S1–S3).

**Figure 1:**
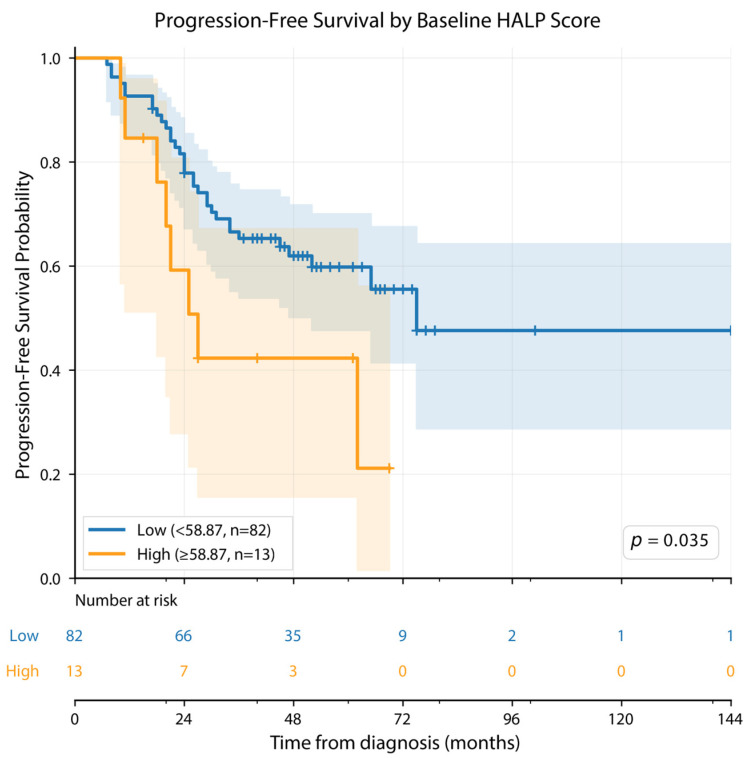
Kaplan–Meier analysis of progression-free survival (PFS) according to baseline HALP score. The cut-off value was determined as 58.87 using the X-tile method. Patients were stratified into a low HALP group (<58.87; *n* = 82, blue) and a high HALP group (≥58.87; *n* = 13, orange). Censored observations are indicated by tick marks (+). Shaded areas represent 95% confidence intervals. Between-group comparison was performed using the log-rank test. The number of patients at risk is shown below the curves at 24-month intervals.

**Figure 2:**
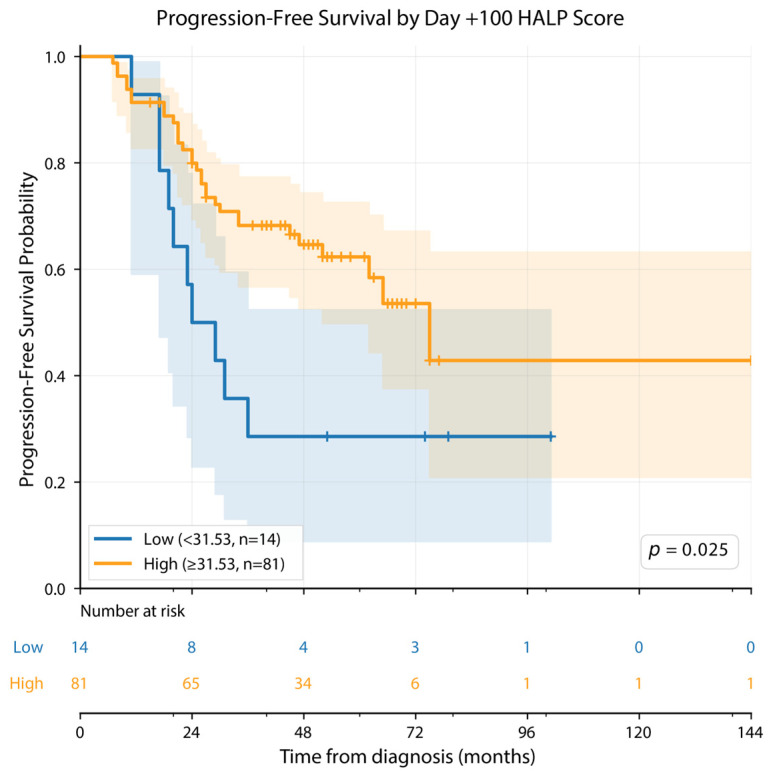
Kaplan–Meier analysis of progression-free survival (PFS) according to HALP score at day +100 post-transplantation. The cut-off value was determined as 31.53 using the X-tile method, optimized for PFS. Patients were stratified into a low HALP group (<31.53; *n* = 14, blue) and a high HALP group (≥31.53; *n* = 81, orange). Censored observations are indicated by tick marks (+). Shaded areas represent 95% confidence intervals. Between-group comparison was performed using the log-rank test. The number of patients at risk is shown below the curves at 24-month intervals.

**Figure 3:**
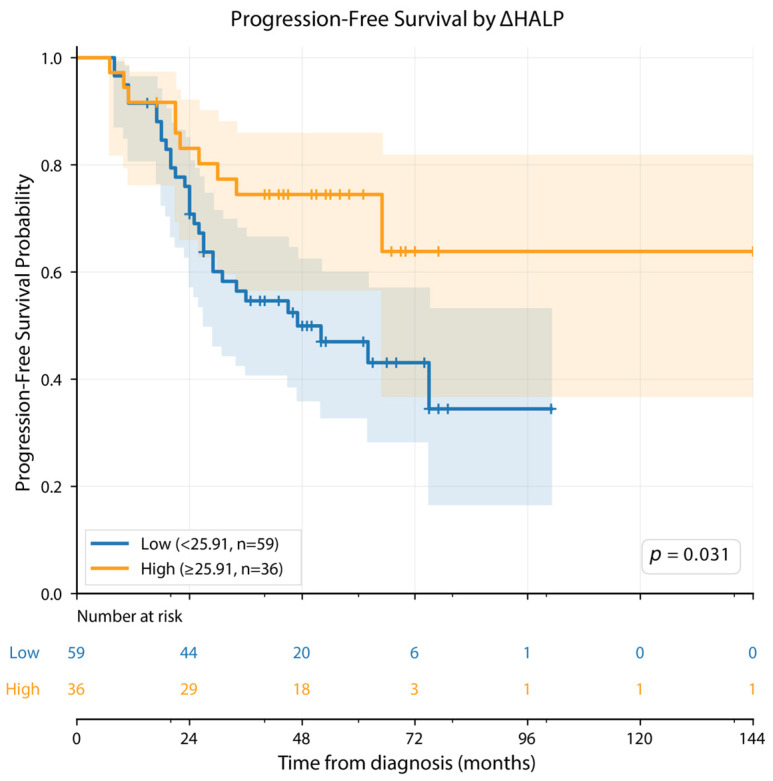
Kaplan–Meier analysis of progression-free survival (PFS) according to ΔHALP. ΔHALP was defined as the difference between HALP at day +100 and baseline HALP. The cut-off value was determined as 25.91 using the X-tile method, optimized for PFS. Patients were stratified into a low ΔHALP group (<25.91; *n* = 59, blue) and a high ΔHALP group (≥25.91; *n* = 36, orange). Censored observations are indicated by tick marks (+). Shaded areas represent 95% confidence intervals. Between-group comparison was performed using the log-rank test. The number of patients at risk is shown below the curves at 24-month intervals.

In contrast, a different pattern was observed for progression-free survival (Table 4, Figure 1, Figure 2 and Figure 3). According to the HALP cut-off at diagnosis, the median PFS was 75.0 months in the low HALP group and 27.0 months in the high HALP group (*p* = 0.035). Based on the day +100 HALP cut-off, the median PFS was 29.0 months in the low HALP group and 75.0 months in the high HALP group (*p* = 0.025). When stratified according to the ΔHALP cut-off, the median PFS was 47.0 months in the low ΔHALP group, whereas the median PFS was not reached in the high ΔHALP group (*p* = 0.031) (Table 4).

In Kaplan–Meier analysis, statistically significant separation in PFS was observed between groups stratified by HALP score at diagnosis, HALP score at day +100, and ΔHALP (log-rank *p* = 0.035, *p* = 0.025, and *p* = 0.031, respectively; Figure 1, Figure 2 and Figure 3).

In multivariable Cox regression analyses adjusting for age, sex, ISS stage, and maintenance therapy, baseline HALP was associated with OS in the unadjusted model; however, this association was substantially attenuated after adjustment for maintenance therapy (Table 5). HALP at day +100 and ΔHALP were not significantly associated with OS. Maintenance therapy itself was the strongest independent predictor of OS across all three models (HR for received vs. not received: 0.19–0.22; all *p* ≤ 0.006).

For PFS, in the unadjusted analyses, baseline HALP, HALP at day +100, ΔHALP, and male sex were associated with PFS. In multivariable models adjusting for maintenance therapy, the inverse association at diagnosis was substantially attenuated (HR for low vs. high: 0.71, 95% CI: 0.28–1.80; *p* = 0.466). In contrast, HALP at day +100 maintained and strengthened its association with PFS after adjustment (HR: 3.27, 95% CI: 1.45–7.38; *p* = 0.004), as did ΔHALP at borderline significance (HR: 1.97; *p* = 0.070). Maintenance therapy was the strongest independent predictor across all three models (HR: 0.16–0.24; all *p* ≤ 0.001) (Table 6).

In multivariable analyses incorporating maintenance therapy, the inverse association between baseline HALP and PFS observed in the unadjusted model was substantially attenuated (HR for low vs. high HALP: 0.71, 95% CI: 0.28–1.80; *p* = 0.466; Table 6). Maintenance therapy itself was the strongest independent predictor of PFS across all three models (HR for received vs. not received: 0.16–0.24; all *p* ≤ 0.001). In contrast, the prognostic association of HALP at day +100 with PFS persisted and strengthened after adjustment for maintenance therapy (HR: 3.27, 95% CI: 1.45–7.38; *p* = 0.004), suggesting that this dynamic parameter captures post-transplant biological information independent of the maintenance treatment effect.

The incremental contribution of dynamic HALP parameters over baseline HALP was assessed using nested Cox models. For PFS, adding HALP at day +100 to the baseline model yielded a statistically significant improvement (χ^2^ = 8.01, df = 1, *p* = 0.005, ΔAIC = −6.01), whereas the addition of ΔHALP also improved model fit (χ^2^ = 4.26, df = 1, *p* = 0.039, ΔAIC = −2.26; Table 7). For OS, neither dynamic parameter showed a statistically significant incremental contribution (HALP at day +100: *p* = 0.085; ΔHALP: *p* = 0.276).

The linearity of the association between HALP scores and survival outcomes was assessed using restricted cubic spline models. None of the six models demonstrated a significant test for non-linearity (all *p* > 0.05), indicating that a linear model was adequate and that no U-shaped relationship was present. In terms of overall model fit, HALP at day +100 showed evidence of an association with survival outcomes in exploratory spline analyses (Table S6). Restricted cubic spline analyses were performed as an exploratory sensitivity analysis to assess potential non-linear associations. Given the limited sample size and number of events, these findings were interpreted descriptively and were not considered confirmatory, and the spline analyses were used to support, rather than to drive, the main Cox regression findings.

In sensitivity analyses, different cut-off strategies were compared. When a literature-based cut-off (28.8) and the cohort median value (32.0) were applied, the HALP score was not associated with survival outcomes (all adjusted *p* > 0.05), highlighting the cut-off-dependent nature of the observed findings. Using the X-tile-derived cut-off (58.87), associations with overall survival and progression-free survival were observed; however, these associations were not consistent across alternative cut-off strategies. Notably, the X-tile-derived threshold was substantially higher than previously reported cut-offs and resulted in a small high-HALP subgroup (*n* = 13) (Table S7). Given the outcome-driven nature of the X-tile approach and the limited size of this subgroup, these findings should be interpreted with caution and considered hypothesis-generating rather than definitive.

## 4. Discussion

In this study, we evaluated the associations of the HALP score at diagnosis, post-transplant day +100, and treatment-related changes (ΔHALP) with survival outcomes in patients with multiple myeloma undergoing autologous stem cell transplantation. Overall, our findings highlight that the prognostic relevance of HALP is not static but depends on the timing of assessment, the clinical endpoint considered, and the broader treatment context. Post-transplant HALP at day +100 emerged as the most robust HALP-based prognostic indicator for PFS, with associations that persisted and strengthened after adjustment for maintenance therapy. In contrast, the inverse association between baseline HALP and PFS was substantially attenuated by maintenance therapy adjustment.

The inverse association between baseline HALP and PFS observed in our cohort warrants careful biological and clinical interpretation. First, supplementary analyses revealed that the high HALP group at diagnosis was characterized by a markedly different hematologic profile: significantly higher lymphocyte counts (median 2890 vs. 1645/µL, *p* < 0.001) and lower platelet counts (median 146,000 vs. 249,000/µL, *p* < 0.001), with comparable hemoglobin and albumin levels (Tables S4 and S8–S11). This pattern is consistent with active disease and bone marrow infiltration, where peripheral lymphocytosis may reflect a reactive immune response or, in some cases, circulating myeloma cells, while reduced platelet counts indicate marrow suppression. Similar paradoxical patterns have been reported in myelodysplastic syndromes [24], where high HALP scores were associated with adverse outcomes—supporting the notion that the prognostic direction of HALP may be disease-specific.

Second, the inverse association may, in part, reflect treatment heterogeneity. In our cohort, patients in the low baseline HALP group were considerably more likely to receive maintenance therapy than those in the high HALP group (88.5% vs. 50.0%; Fisher’s exact *p* = 0.004). Incorporation of maintenance therapy into the multivariable Cox model substantially attenuated the HALP–PFS association (HR shifted from 0.34 to 0.71). This suggests that the apparent prognostic advantage of low baseline HALP at the univariable level may be partly mediated through differential access to maintenance therapy, with maintenance itself emerging as the strongest independent predictor of PFS in our cohort. The persistence and strengthening of the HALP at day +100 association after adjustment for maintenance underscores the distinct biological information captured by this post-transplant timepoint.

In our cohort, baseline HALP was independently associated with both OS and PFS in multivariable analyses. These findings are consistent with previous reports in multiple myeloma, including the studies by Solmaz et al. [19] and Zhang et al. [20], although differences in study design, cut-off determination, and transplant status should be considered when interpreting cross-study comparisons. In addition, a large meta-analysis in solid tumors demonstrated an overall association between low HALP scores and adverse survival outcomes [25], supporting the broader prognostic relevance of HALP in oncologic settings.

A key finding of this study is the differential prognostic relevance of baseline versus dynamic HALP parameters. While baseline HALP reflects pre-treatment immunonutritional status, post-transplant HALP and ΔHALP may better capture treatment-related biological changes, particularly immune reconstitution and nutritional recovery following ASCT. This dynamic behavior may explain the stronger and more consistent associations observed with progression-free survival. Therefore, HALP should not be interpreted as a static biomarker, but rather as a time-dependent indicator reflecting evolving host–disease interactions throughout the treatment course.

In classical Hodgkin lymphoma, HALP has not consistently demonstrated independent prognostic significance [26]. In line with this disease-specific behavior, Zhang et al. [27] recently examined the lymphocyte-to-monocyte index and HALP score in 350 patients with monoclonal gammopathy of undetermined significance (MGUS); while the lymphocyte-to-monocyte index was associated with MGUS prevalence, the HALP score did not show a significant association—further supporting the heterogeneous behavior of HALP across different stages of the plasma cell dyscrasia spectrum. These findings collectively suggest that HALP may reflect disease-specific immunonutritional and inflammatory dynamics rather than a universally consistent biological effect.

In our cohort, all patients received triplet-based induction therapy. Notably, only two patients (2.1%) received daratumumab-containing (anti-CD38) induction regimens, as these regimens were not yet routinely available at our institution during the early years of the study period. The findings should therefore be interpreted within the context of predominantly triplet-based induction.

The evaluation of HALP at post-transplant day 100 and its dynamic change during treatment represents an important aspect of this study. Associations of day-100 HALP and ΔHALP were observed primarily with PFS, suggesting that post-transplant immunonutritional status may be more closely related to short- to mid-term disease control. In contrast, overall survival is likely influenced by additional factors, including disease biology and subsequent lines of therapy.

The limited ability of day +100 HALP to predict treatment response (AUC values with 95% confidence intervals encompassing 0.50; Table S12) suggests that immunonutritional indices may be more closely related to long-term disease trajectory rather than immediate treatment sensitivity. Treatment response is influenced by multiple factors, including cytogenetic risk, disease burden, and treatment-related variables, which may not be fully captured by HALP alone. Accordingly, the previous claim that HALP provides “complementary prognostic information” for response prediction has been moderated; the value of HALP in this cohort appears to lie in its association with PFS rather than in response classification.

Notably, the composite HALP score appeared to provide more informative associations with survival outcomes than serum albumin alone. Although albumin is incorporated into both ISS and HALP, albumin alone did not demonstrate consistent associations in the alternative specification (Model B; Table S13), whereas HALP-based models showed more stable associations. Similar findings have been reported for other composite indices such as NRI and CONUT in multiple myeloma [28,29,30], supporting the potential value of integrated immunonutritional biomarkers.

The prognostic direction of the HALP score is not uniform across malignancies, and understanding why both high and low HALP values may be associated with adverse outcomes in different disease contexts is essential for interpreting our findings correctly. In most solid tumors and several hematologic malignancies, low HALP is consistently associated with worse outcomes, reflecting its role as a composite marker of poor nutritional reserve, systemic inflammation, and impaired immune competence. In diffuse large B-cell lymphoma, lower HALP scores have been independently associated with advanced disease stage, suboptimal treatment response, and significantly worse OS and event-free survival in two separate cohorts, with cut-off values of 20.8 and 26.17, respectively, and these associations persisted after adjustment for established prognostic indices including the International Prognostic Index [17,18]. Similarly, in extranodal natural killer/T-cell lymphoma, a HALP score below 32.6 was independently associated with poorer clinicopathological characteristics including worse Eastern Cooperative Oncology Group performance status, B symptoms, elevated lactate dehydrogenase, and suboptimal response, and was linked to significantly lower 5-year OS and PFS in both the PINK and PINK-E risk models [31]. This low-HALP-is-adverse paradigm also holds in MM when conventional cut-offs are applied: Solmaz et al. [19] reported significantly longer OS in patients with HALP above 28.8, with the score retaining independent prognostic significance alongside NLR, PLR, and ISS stage in multivariate analysis, while lower HALP and lower Geriatric Nutritional Risk Index were jointly associated with shorter OS and disease-free survival in diffuse large B cell lymphoma [18].

However, this directional consistency does not extend to all hematologic malignancies. In classical Hodgkin lymphoma, HALP has demonstrated inconsistent prognostic behavior: while high HALP was significantly associated with early-stage favorable disease in one cohort [26], another study found that the HALP score lost its association with PFS in multivariate analysis and showed no significant association with OS, with systemic immune-inflammation-related indices such as Systemic Inflammation Response Index emerging as stronger independent predictors [26,32]. These observations suggest that in diseases where treatment has profoundly improved survival—such as classical Hodgkin lymphoma in the immunotherapy era—the incremental discriminatory contribution of immunonutritional indices like HALP may be attenuated by the dominant effect of modern therapy [26]. Most strikingly, in myelodysplastic syndromes—a bone marrow-infiltrating hematologic malignancy sharing key pathobiological features with MM—the prognostic direction of HALP is paradoxically reversed: higher HALP scores were associated with intermediate- and high-risk disease under IPSS and IPSS-R classifications, and patients with HALP above 67.5 had significantly lower survival rates, with 3-, 5-, and 10-year survival rates of only 25%, 18%, and 11%, respectively [24]. This inversion has the same mechanistic explanation we propose for our MM cohort: in bone marrow-infiltrating diseases, thrombocytopenia driven by marrow suppression and lymphocytosis reflecting reactive or clonal immune dysregulation mathematically inflate the HALP score in ways that reflect disease burden rather than host fitness, decoupling the score from its intended immunonutritional interpretation [24]. Collectively, these cross-disease observations support the conclusion that the HALP score does not carry a fixed prognostic direction, and that its biological meaning—and therefore the cut-off values, directionality, and clinical applicability—must be interpreted within the specific disease context, the timing of assessment, and the treatment landscape in which it is evaluated.

### Strengths and Limitations

The strengths of this study include the evaluation of HALP at multiple clinically relevant timepoints, the assessment of cut-off-dependent behavior, the use of restricted cubic spline analyses to explore potential non-linearity, sensitivity analyses employing alternative cut-off strategies, the formal evaluation of incremental prognostic contribution using nested Cox models with the likelihood ratio test, and the incorporation of maintenance therapy into the multivariable models.

Several limitations should be acknowledged. First, the retrospective single-center design limits generalizability and introduces the possibility of selection and information bias. Second, the relatively small sample size and limited number of events—particularly for OS (22 deaths; events-per-variable = 4.2 in models with five covariates)—fall below conventional EPV thresholds. Accordingly, OS findings should be regarded as exploratory and hypothesis-generating rather than definitive. Third, the use of outcome-driven X-tile cut-offs in the same dataset used for outcome estimation introduces a recognized risk of optimism bias, and the absence of external validation precludes generalization of the specific cut-off values to other cohorts. Fourth, cytogenetic risk profiles (del[17p], t[4;14], t[14;16], 1q21 amplification) and minimal residual disease (MRD) assessments could not be incorporated into the analyses, as these data were not systematically available in the institutional records during the study period. The omission of these established prognostic factors limits the interpretation of HALP’s independent contribution and should be addressed in future prospective studies. Fifth, the response prediction analyses were limited by class imbalance (16 of 95 patients in the suboptimal-response group), and the AUC values (0.48–0.58) with 95% confidence intervals encompassing 0.50 indicate that HALP cannot be recommended for individual response prediction. Sixth, although maintenance therapy was incorporated into the multivariable models, residual confounding from unmeasured factors (e.g., performance status, comorbidities, and post-relapse therapies) cannot be excluded.

## 5. Conclusions

In this single-center retrospective cohort, post-transplant HALP at day +100 emerged as the most robust HALP-based prognostic indicator for progression-free survival, with associations that persisted and strengthened after adjustment for maintenance therapy. In contrast, the association between baseline HALP and PFS was substantially attenuated after maintenance therapy adjustment, and the discriminative performance of HALP for treatment response prediction was limited (with all 95% confidence intervals encompassing AUC = 0.50).

Given its simplicity and accessibility, HALP—particularly when assessed dynamically at post-transplant day +100—may warrant further evaluation as a potential complementary biomarker for risk stratification. However, the present findings should be regarded as exploratory and hypothesis-generating in light of the small sample size, the limited number of OS events, the use of outcome-driven cut-offs without external validation, and the absence of cytogenetic and minimal residual disease data. Validation in larger, prospective, multi-center cohorts is required before any clinical implementation can be considered.

## Figures and Tables

**Table 1:** Baseline characteristics of patients.

Variables	*n* = 95
Age (years), median [range]	65.0 [42.0–75.0]
Sex	
Female	38 (40.0)
Male	57 (60.0)
ISS Stage	
Stage I	50 (52.6)
Stage II	26 (27.4)
Stage III	19 (20.0)
Myeloma subtype	
IgG kappa	31 (32.6)
IgG lambda	26 (27.4)
IgA kappa	11 (11.6)
IgA lambda	8 (8.4)
Lambda light chain	7 (7.4)
Kappa light chain	5 (5.3)
Non-secretory	7 (7.4)
Time from diagnosis to transplant (months), median [range]	7.3 [2.0–75.0]
Induction regimens *	
VCD-based	80 (84.2)
VRD-based	23 (24.2)
VAD-based	15 (15.8)
KRD-based	15 (15.8)
VTD-based	3 (3.2)
Daratumumab-based (anti-CD38)	2 (2.1)
Day +100 treatment response	
Stringent complete response (sCR) †	25 (26.3)
Very good partial response (VGPR)	54 (56.8)
Partial response (PR)	12 (12.6)
Stable disease (SD)	2 (2.1)
Progressive disease (PD)	2 (2.1)
Maintenance therapy	
Received maintenance	75 (78.9)
No maintenance	15 (15.8)
Unknown	5 (5.3)
Maintenance regimen (among receivers)	
Lenalidomide monotherapy	56 (74.7)
Combination regimens	11 (14.7)
Other agents	8 (10.7)
Maintenance duration (months), median [range]	41 [14–140]

Data are presented as *n* (%) for categorical variables and median [minimum–maximum] for non-normally distributed continuous variables. ISS: International Staging System; sCR: Stringent Complete Response; VGPR: Very Good Partial Response; PR: Partial Response; SD: Stable Disease; PD: Progressive Disease; VCD: Bortezomib, Cyclophosphamide, Dexamethasone; VRD: Bortezomib, Lenalidomide, Dexamethasone; VAD: Vincristine, Doxorubicin, Dexamethasone; KRD: Carfilzomib, Lenalidomide, Dexamethasone; VTD: Bortezomib, Thalidomide, Dexamethasone. * Some patients received more than one induction regimen during the pre-transplant period; therefore, percentages may exceed 100%. † Following clarification with the treating clinician, all patients initially recorded as complete response (CR) fulfilled stringent complete response (sCR) criteria according to IMWG.

**Table 2:** Distribution of laboratory parameters at diagnosis and day-100 post-transplant.

Laboratory Parameters	At Diagnosis	Day-100
Hemoglobin (g/dL)	10.54 ± 2.27	12.27 ± 1.41
Albumin (g/dL)	3.73 ± 0.67	4.40 [3.03–5.10]
Lymphocyte (×10^3^/µL)	1.88 [0.63–18.50]	1.73 [0.54–4.79]
Platelet (×10^3^/µL)	236.0 [32.0–614.0]	183.69 ± 60.95
HALP score	32.03 [3.48–468.74]	57.83 [8.58–831.66]
ΔHALP (Day-100 − Diagnosis)	19.47 [−389.77–804.81]	

Data are presented as mean ± standard deviation for normally distributed continuous variables and median [minimum–maximum] for non-normally distributed continuous variables. Normality was assessed using Shapiro–Wilk test. HALP score was calculated using predefined laboratory parameters. ΔHALP: Change in HALP (Day-100 HALP score − Baseline HALP score).

**Table 3:** Optimal HALP cut-off points for survival outcomes determined by X-tile method.

HALP Variable	Outcome	Optimal Cut-Off	χ^2^ (Log-Rank)	*p*
HALP (diagnosis)	OS	58.87	3.01	0.083
	PFS	58.87	4.45	**0.035**
HALP (day-100)	OS	75.52	1.10	0.294
	PFS	31.53	5.05	**0.025**
ΔHALP	OS	42.10	2.14	0.143
	PFS	25.91	5.58	**0.018**

Optimal cut-off points were determined using the X-tile algorithm, which maximizes the log-rank statistic. The χ^2^ and *p* values reported here reflect the X-tile optimization statistic at the maximum log-rank threshold and are upwardly biased estimates; the corresponding standard log-rank *p* values from Kaplan–Meier analysis are reported in Table 4. Given the outcome-driven nature of the X-tile approach and the limited size of this subgroup, these findings should be interpreted with caution and considered hypothesis-generating rather than definitive. Bold *p*-values indicate statistical significance (*p* ≤ 0.05). OS: Overall Survival; PFS: Progression-Free Survival; HALP: Hemoglobin × Albumin × Lymphocyte/Platelet; ΔHALP: Change in HALP.

**Table 4:** Comparison of progression-free survival by HALP score groups using Kaplan–Meier method.

Variables	*n*	Events (Progression/Death)	Median Survival (Months)	*p* (Log-Rank)
Progression-Free Survival				
HALP at diagnosis				
Low (<58.87)	82	33	75.0	**0.035**
High (≥58.87)	13	8	27.0	
HALP at day +100				
Low (<31.53)	14	10	29.0	**0.025**
High (≥31.53)	81	31	75.0	
ΔHALP				
Low (<25.91)	59	31	47.0	**0.031**
High (≥25.91)	36	10	NR	
Overall Survival				
HALP at diagnosis				
Low (<58.87)	82	17	123.0	0.083
High (≥58.87)	13	5	NR	
HALP at day +100				
Low (<75.52)	68	18	123.0	0.294
High (≥75.52)	27	4	NR	
ΔHALP				
Low (<42.10)	70	19	123.0	0.143
High (≥42.10)	25	3	NR	

Cut-off points were optimized using the X-tile method for each outcome. Between-group comparisons were performed using the log-rank test. NR: Not Reached (median survival time was not reached). HALP: Hemoglobin × Albumin × Lymphocyte/Platelet; ΔHALP: Change in HALP. Bold *p*-values indicate statistical significance (*p* ≤ 0.05).

**Table 5:** Univariate and multivariate cox regression analysis of prognostic factors for overall survival.

Variables	Univariate HR [95% CI]	*p*	Multivariate HR [95% CI]	*p*
Model 1: HALP at diagnosis				
HALP at diagnosis (low vs. high)	0.51 [0.17–1.53]	0.232	0.67 [0.18–2.42]	0.539
Age (years)	0.99 [0.93–1.04]	0.615	1.00 [0.95–1.06]	0.902
Sex (male vs. female)	0.45 [0.19–1.09]	0.076	0.37 [0.14–0.98]	**0.045**
ISS Stage (III vs. I–II)	1.50 [0.54–4.14]	0.437	2.63 [0.86–8.07]	0.091
Maintenance therapy (yes vs. no)	0.25 [0.10–0.61]	**0.002**	0.22 [0.07–0.65]	**0.006**
Model 2: HALP at day +100				
HALP at day +100 (low vs. high)	1.82 [0.61–5.42]	0.285	1.53 [0.51–4.64]	0.451
Age (years)	0.99 [0.93–1.04]	0.615	1.01 [0.95–1.06]	0.831
Sex (male vs. female)	0.45 [0.19–1.09]	0.076	0.42 [0.17–1.07]	0.070
ISS Stage (III vs. I–II)	1.50 [0.54–4.14]	0.437	2.65 [0.86–8.13]	0.089
Maintenance therapy (yes vs. no)	0.25 [0.10–0.61]	**0.002**	0.19 [0.07–0.53]	**0.001**
Model 3: ΔHALP				
ΔHALP (low vs. high)	2.47 [0.72–8.42]	0.150	1.84 [0.51–6.59]	0.349
Age (years)	0.99 [0.93–1.04]	0.615	1.01 [0.96–1.07]	0.729
Sex (male vs. female)	0.45 [0.19–1.09]	0.076	0.42 [0.17–1.05]	0.062
ISS Stage (III vs. I–II)	1.50 [0.54–4.14]	0.437	2.73 [0.88–8.41]	0.081
Maintenance therapy (yes vs. no)	0.25 [0.10–0.61]	**0.002**	0.21 [0.08–0.58]	**0.003**

Multivariate model includes the HALP variable, age, sex, ISS stage, and maintenance therapy. The analysis was restricted to patients with known maintenance therapy status (*n* = 90, 21 OS events). Cut-off points were optimized for OS using the X-tile method (HALP at diagnosis: 58.87; HALP at day +100: 75.52; ΔHALP: 42.10). Proportional hazards assumption was verified using Schoenfeld residuals. HR < 1 indicates lower risk in the low HALP group. Given the limited number of OS events (events-per-variable = 4.2), multivariate findings should be interpreted as exploratory. HR: Hazard Ratio; CI: Confidence Interval; ISS: International Staging System; HALP: Hemoglobin × Albumin × Lymphocyte/Platelet; ΔHALP: Change in HALP. Bold *p*-values indicate statistical significance (*p* ≤ 0.05).

**Table 6:** Univariate and multivariate cox regression analysis of prognostic factors for progression-free survival.

Variables	Univariate HR [95% CI]	*p*	Multivariate HR [95% CI]	*p*
Model 1: HALP at diagnosis				
HALP at diagnosis (low vs. high)	0.49 [0.21–1.11]	0.088	0.71 [0.28–1.80]	0.466
Age (years)	0.98 [0.94–1.02]	0.356	0.99 [0.95–1.03]	0.552
Sex (male vs. female)	0.47 [0.25–0.89]	**0.020**	0.38 [0.19–0.74]	**0.005**
ISS Stage (III vs. I–II)	1.40 [0.66–2.98]	0.376	2.40 [1.04–5.51]	**0.040**
Maintenance therapy (yes vs. no)	0.27 [0.13–0.57]	**<0.001**	0.24 [0.10–0.57]	**0.001**
Model 2: HALP at day +100				
HALP at day +100 (low vs. high)	2.01 [0.95–4.26]	0.068	3.27 [1.45–7.38]	**0.004**
Age (years)	0.98 [0.94–1.02]	0.356	0.99 [0.95–1.03]	0.654
Sex (male vs. female)	0.47 [0.25–0.89]	**0.020**	0.34 [0.17–0.66]	**0.002**
ISS Stage (III vs. I–II)	1.40 [0.66–2.98]	0.376	2.38 [1.04–5.45]	**0.040**
Maintenance therapy (yes vs. no)	0.27 [0.13–0.57]	**<0.001**	0.16 [0.07–0.37]	**<0.001**
Model 3: ΔHALP				
ΔHALP (low vs. high)	2.19 [1.07–4.50]	**0.033**	1.97 [0.95–4.08]	0.070
Age (years)	0.98 [0.94–1.02]	0.356	0.99 [0.95–1.03]	0.590
Sex (male vs. female)	0.47 [0.25–0.89]	**0.020**	0.41 [0.21–0.80]	**0.009**
ISS Stage (III vs. I–II)	1.40 [0.66–2.98]	0.376	2.45 [1.07–5.59]	**0.033**
Maintenance therapy (yes vs. no)	0.27 [0.13–0.57]	**<0.001**	0.23 [0.10–0.51]	**<0.001**

Multivariate model includes the HALP variable, age, sex, ISS stage, and maintenance therapy. The analysis was restricted to patients with known maintenance therapy status (*n* = 90, 39 PFS events; events-per-variable = 7.8). Cut-off points were optimized for PFS using the X-tile method (HALP at diagnosis: 58.87; HALP at day +100: 31.53; ΔHALP: 25.91). Proportional hazards assumption was verified using Schoenfeld residuals. HR < 1 indicates lower risk in the low HALP group (or yes vs. no for binary covariates). HR: Hazard Ratio; CI: Confidence Interval; ISS: International Staging System; HALP: Hemoglobin × Albumin × Lymphocyte/Platelet; ΔHALP: Change in HALP. Bold *p*-values indicate statistical significance (*p* ≤ 0.05).

**Table 7:** Likelihood ratio test for the incremental prognostic contribution of dynamic HALP parameters over baseline HALP.

Comparisons	χ^2^	df	*p*	ΔAIC
Outcome: Progression-Free Survival				
HALP at day +100 added to baseline HALP	8.01	1	0.0047	−6.01
ΔHALP added to baseline HALP	4.26	1	0.0391	−2.26
Outcome: Overall Survival				
HALP at day +100 added to baseline HALP	2.97	1	0.0848	−0.97
ΔHALP added to baseline HALP	1.19	1	0.2762	+0.81

Nested Cox proportional hazards models were compared using the likelihood ratio test. The base model included baseline HALP (low vs. high), age, sex, and ISS stage. Each dynamic HALP parameter (HALP at day +100 or ΔHALP, low vs. high) was added separately. A negative ΔAIC favors the more complex (full) model. χ^2^: chi-square statistic; df: degrees of freedom; ΔAIC: change in Akaike Information Criterion (full minus base); HALP: Hemoglobin × Albumin × Lymphocyte/Platelet; ΔHALP: Change in HALP.

## Data Availability

The data presented in this study are available from the corresponding author upon reasonable request. The data are not publicly available due to privacy and ethical restrictions.

## Supplementary Materials

The following supporting information can be downloaded at: https://www.mdpi.com/article/10.3390/cancers18101570/s1, Table S1: Comparison of HALP scores by sex, ISS stage, treatment response, and survival status; Table S2: Evaluation of the correlation between HALP scores and age; Table S3: ROC analysis of HALP scores for predicting day +100 treatment response; Table S4: Comparison of hemoglobin, albumin, lymphocyte, and platelet levels between low and high HALP groups stratified by HALP at diagnosis; Table S5: Comparison of overall survival by HALP score groups using the Kaplan–Meier method; Table S6: Evaluation of the linearity of the relationship between HALP scores and survival using restricted cubic spline Cox models; Table S7: Sensitivity analysis comparing the prognostic value of the HALP score according to different cut-off points; Table S8: Same comparison stratified by HALP at day +100 (PFS-optimized cut-off); Table S9: Same comparison stratified by HALP at day +100 (OS-optimized cut-off); Table S10: Same comparison stratified by ΔHALP (PFS-optimized cut-off); Table S11: Same comparison stratified by ΔHALP (OS-optimized cut-off); Table S12: Diagnostic performance of HALP score variables for predicting day +100 treatment response (AUC with bootstrap 95% confidence intervals); Table S13: Multivariate Cox regression with serum albumin in place of HALP (Model B alternative specification); Figure S1: Kaplan–Meier analysis of overall survival according to HALP at diagnosis (with number-at-risk); Figure S2: Kaplan–Meier analysis of overall survival according to HALP at day +100 (with number-at-risk); Figure S3: Kaplan–Meier analysis of overall survival according to ΔHALP (with number-at-risk); Figure S4: Receiver operating characteristic (ROC) curves evaluating the diagnostic performance of HALP score variables (HALP at diagnosis, HALP at day +100, and ΔHALP) for predicting day +100 treatment response (good response: sCR + VGPR vs. suboptimal response: PR + SD + PD).
