# Supplementary material for: Dynamic HALP Score as a Time-Dependent Prognostic Biomarker in Multiple Myeloma Patients Undergoing Autologous Stem Cell Transplantation
Source: Cancers (Basel). 2026 May 12;18(10):1570. doi: 10.3390/cancers18101570 (PMC13204590; doi:10.3390/cancers18101570)
Supplement: Supplementary file 1 [file cancers-18-01570-s001.zip › cancers-4257166-supplementary.pdf]

# Supplementary Materials

## Supplementary Tables

**Table S1.** Comparison of HALP scores by sex, ISS stage, treatment response, and survival status.

| HALP Variables | Group 1 (n) | Median [IQR] | Group 2 (n) | Median [IQR] | Group 3 (n) | Median [IQR] | p |
|---|---|---|---|---|---|---|---|
| **Sex** | | | | | | | |
| HALP at diagnosis | Female (n=38) | 26.13 [18.39–36.72] | Male (n=57) | 37.87 [22.08–52.36] | — | — | **0.011** |
| HALP at day +100 | Female (n=38) | 41.30 [36.53–76.02] | Male (n=57) | 62.36 [41.34–81.55] | — | — | 0.091 |
| ΔHALP | Female (n=38) | 19.67 [10.61–39.63] | Male (n=57) | 18.92 [6.97–43.00] | — | — | 0.823 |
| **ISS Stage** | | | | | | | |
| HALP at diagnosis | Stage I (n=50) | 35.64 [22.50–47.70] | Stage II (n=26) | 28.58 [20.48–44.53] | Stage III (n=19) | 22.72 [16.68–38.96] | 0.204 |
| HALP at day +100 | Stage I (n=50) | 47.82 [36.19–77.12] | Stage II (n=26) | 65.67 [46.38–81.17] | Stage III (n=19) | 57.83 [34.28–79.11] | 0.156 |
| ΔHALP | Stage I (n=50) | 15.32 [5.68–29.16] | Stage II (n=26) | 32.16 [15.45–43.64] | Stage III (n=19) | 20.95 [10.23–49.50] | **0.043** |
| **Day +100 response** | | | | | | | |
| HALP at diagnosis | Good (sCR + VGPR) (n=79) | 30.66 [20.19–45.94] | Other (PR + SD + PD) (n=16) | 33.20 [26.50–49.88] | — | — | 0.337 |
| HALP at day +100 | Good (sCR + VGPR) (n=79) | 57.83 [36.88–78.13] | Other (PR + SD + PD) (n=16) | 61.16 [38.92–77.87] | — | — | 0.831 |
| ΔHALP | Good (sCR + VGPR) (n=79) | 18.92 [7.77–42.96] | Other (PR + SD + PD) (n=16) | 19.63 [8.87–37.90] | — | — | 0.800 |
| **Progression/Relapse** | | | | | | | |
| HALP at diagnosis | No (n=54) | 30.69 [20.28–44.17] | Yes (n=41) | 33.11 [20.63–47.89] | — | — | 0.390 |
| HALP at day +100 | No (n=54) | 60.06 [39.31–83.92] | Yes (n=41) | 44.55 [34.13–72.74] | — | — | 0.112 |
| ΔHALP | No (n=54) | 23.42 [9.55–47.92] | Yes (n=41) | 18.49 [5.25–23.07] | — | — | 0.071 |
| **Death** | | | | | | | |
| HALP at diagnosis | Alive (n=73) | 32.03 [20.26–45.95] | Deceased (n=22) | 32.27 [23.02–51.24] | — | — | 0.404 |
| HALP at day +100 | Alive (n=73) | 57.98 [36.85–80.42] | Deceased (n=22) | 55.62 [40.23–72.15] | — | — | 0.801 |
| ΔHALP | Alive (n=73) | 20.17 [7.86–43.46] | Deceased (n=22) | 16.60 [7.50–33.28] | — | — | 0.390 |

Group comparisons were performed using the Mann–Whitney U test for two-group comparisons and the Kruskal–Wallis test for three-group comparisons (ISS stage). Data are presented as median [interquartile range, 25th–75th percentile]. Bold p-values indicate statistical significance ($p \leq 0.05$). HALP: Hemoglobin × Albumin × Lymphocyte / Platelet; ΔHALP: Change in HALP; ISS: International Staging System; sCR: Stringent Complete Response; VGPR: Very Good Partial Response; PR: Partial Response; SD: Stable Disease; PD: Progressive Disease.

**Table S2.** Evaluation of the correlation between HALP scores and age.

| HALP Variable | n | Spearman's r | p |
|---|---|---|---|
| HALP at diagnosis | 95 | -0.001 | 0.994 |
| HALP at day +100 | 95 | +0.120 | 0.248 |
| ΔHALP | 95 | +0.063 | 0.543 |

Correlations between HALP score variables and patient age were assessed using Spearman's rank correlation coefficient. A positive r indicates higher HALP values with increasing age; a negative r indicates the opposite. None of the three HALP variables were significantly correlated with age (all $p > 0.05$). HALP: Hemoglobin × Albumin × Lymphocyte / Platelet; ΔHALP: Change in HALP.

**Table S3.** ROC analysis of HALP scores for predicting day +100 treatment response.

| HALP Variable | AUC | 95% CI | p |
|---|---|---|---|
| HALP at diagnosis | 0.577 | 0.437–0.728 | 0.344 |
| HALP at day +100 | 0.517 | 0.355–0.690 | 0.828 |
| ΔHALP | 0.479 | 0.329–0.629 | 0.798 |

The dependent variable was day +100 treatment response, dichotomized as good response (sCR + VGPR; n = 79) versus other responses (PR + SD + PD; n = 16). The 95% confidence interval was estimated using non-parametric bootstrap resampling (1,000 replications). None of the three HALP variables demonstrated significant discriminative ability (all $p > 0.05$). AUC: Area Under the Receiver Operating Characteristic Curve; CI: Confidence Interval; HALP: Hemoglobin × Albumin × Lymphocyte / Platelet; ΔHALP: Change in HALP.

**Table S4.** Comparison of Hemoglobin, Albumin, Lymphocyte, and Platelet Levels Between Low and High HALP Groups — HALP at diagnosis (cut-off 58.87).

| Biomarker | Low n | Low Median [IQR] | High n | High Median [IQR] | p | r |
|---|---|---|---|---|---|---|
| Hemoglobin (g/dL) at diagnosis | n=82 | 10.50 [8.60–12.10] | n=13 | 12.00 [9.00–12.50] | 0.2836 | +0.187 |
| Albumin (g/dL) at diagnosis | n=82 | 3.90 [3.32–4.20] | n=13 | 3.80 [3.10–3.90] | 0.2245 | -0.211 |
| Lymphocyte (×10³/µL) at diagnosis | n=82 | 1645.00 [1272.50–2285.00] | n=13 | 2890.00 [2350.00–3040.00] | <0.0001 | +0.706 |
| Platelet (×10³/µL) at diagnosis | n=82 | 249000.00 [186250.00–293750.00] | n=13 | 146000.00 [92000.00–183000.00] | <0.0001 | -0.693 |

Group comparisons were performed using the Mann–Whitney U test. The rank-biserial correlation coefficient (r) is reported as an effect size measure, calculated as $r = 1 - (2U)/(n_1 \times n_2)$. Patients were stratified using the X-tile-derived cut-off (58.87) for HALP at diagnosis. The significantly higher lymphocyte count and lower platelet count observed in the high HALP group are consistent with active disease and bone marrow infiltration in multiple myeloma. HALP: Hemoglobin × Albumin × Lymphocyte / Platelet; IQR: Interquartile Range.

**Table S5.** Comparison of overall survival by HALP score groups using the Kaplan–Meier method.

| **Variables** | **n** | **Events (Death)** | **Median OS (Months)** | **p (Log-Rank)** |
|---|---|---|---|---|
| **HALP at diagnosis** | | | | |
| Low (<58.87) | 82 | 17 | 123.0 | 0.083 |
| High (≥58.87) | 13 | 5 | NR | |
| **HALP at day +100** | | | | |
| Low (<75.52) | 68 | 18 | 123.0 | 0.294 |
| High (≥75.52) | 27 | 4 | NR | |
| **ΔHALP** | | | | |
| Low (<42.10) | 70 | 19 | 123.0 | 0.143 |
| High (≥42.10) | 25 | 3 | NR | |

Cut-off points were optimized for OS using the X-tile method (HALP at diagnosis: 58.87; HALP at day +100: 75.52; ΔHALP: 42.10). Between-group comparisons were performed using the log-rank test. None of the three HALP variables demonstrated statistically significant separation for OS (all $p > 0.05$). NR: Not Reached; OS: Overall Survival; HALP: Hemoglobin × Albumin × Lymphocyte / Platelet; ΔHALP: Change in HALP.

**Table S6.** Evaluation of the linearity of the relationship between HALP scores and survival outcomes using restricted cubic spline Cox models.

| HALP Variable | Outcome | p (Overall Model) | p (Non-Linearity) |
|---|---|---|---|
| HALP at diagnosis | OS | 0.108 | 0.162 |
| HALP at diagnosis | PFS | 0.032 | 0.110 |
| HALP at day +100 | OS | 0.058 | 0.335 |
| HALP at day +100 | PFS | 0.028 | 0.082 |
| ΔHALP | OS | 0.222 | 0.108 |
| ΔHALP | PFS | 0.078 | 0.076 |

Restricted cubic spline analyses were performed as exploratory sensitivity analyses to assess potential non-linear associations. The "Overall Model" p-value reflects the joint significance of the spline terms (likelihood ratio test against the null model), while the "Non-Linearity" p-value tests whether the non-linear (log-transformed) component improved fit beyond a linear specification. None of the six models demonstrated a statistically significant departure from linearity (all $p > 0.05$), indicating that a linear model was adequate. HALP: Hemoglobin × Albumin × Lymphocyte / Platelet; ΔHALP: Change in HALP; OS: Overall Survival; PFS: Progression-Free Survival.

**Table S7.** Sensitivity analysis comparing the prognostic value of the baseline HALP score according to different cut-off points.

| Outcome | n (Low / High) | Univariate HR [95% CI] | p | Multivariate HR [95% CI] | p |
|---|---|---|---|---|---|
| **Cut-off = 28.8 (literature-based, Solmaz et al.)** | | | | | |
| OS | 39 / 51 | 1.13 [0.48–2.69] | 0.775 | 1.57 [0.61–4.06] | 0.349 |
| PFS | 39 / 51 | 1.20 [0.63–2.27] | 0.577 | 1.53 [0.78–2.97] | 0.214 |
| **Cut-off = 32.0 (cohort median)** | | | | | |
| OS | 44 / 46 | 0.93 [0.39–2.19] | 0.868 | 1.21 [0.47–3.11] | 0.688 |
| PFS | 44 / 46 | 1.24 [0.66–2.33] | 0.507 | 1.56 [0.81–3.01] | 0.181 |
| **Cut-off = 58.87 (X-tile–derived)** | | | | | |
| OS | 78 / 12 | 1.95 [0.65–5.85] | 0.232 | 1.50 [0.41–5.44] | 0.539 |
| PFS | 78 / 12 | 2.05 [0.90–4.67] | 0.088 | 1.42 [0.56–3.61] | 0.466 |

Sensitivity analyses comparing three cut-off strategies for baseline HALP: a literature-based value (28.8) previously reported in a Turkish multiple myeloma cohort by Solmaz et al. [19], the cohort median value (32.0), and the X-tile–derived value (58.87). Multivariate models adjusted for age, sex, ISS stage (III vs. I–II), and maintenance therapy (n = 90 with known maintenance status). HR < 1 indicates lower risk in the low HALP group. When literature-based and cohort-median cut-offs were applied, no significant associations with OS or PFS were observed in either univariate or multivariate models, highlighting the cut-off-dependent nature of the X-tile findings. HR: Hazard Ratio; CI: Confidence Interval; OS: Overall Survival; PFS: Progression-Free Survival; ISS: International Staging System; HALP: Hemoglobin × Albumin × Lymphocyte / Platelet. Bold p-values indicate statistical significance ($p \leq 0.05$).

**Table S8.** Comparison of Hemoglobin, Albumin, Lymphocyte, and Platelet Levels Between Low and High HALP Groups — HALP at day +100, PFS-optimized (cut-off 31.53).

| Biomarker | Low n | Low Median [IQR] | High n | High Median [IQR] | p | r |
|---|---|---|---|---|---|---|
| Hemoglobin (g/dL) at day +100 | n=14 | 11.35 [10.43–12.25] | n=81 | 12.30 [11.60–13.20] | 0.0200 | +0.392 |
| Albumin (g/dL) at day +100 | n=14 | 4.30 [4.03–4.47] | n=81 | 4.50 [4.20–4.60] | 0.1671 | +0.232 |
| Lymphocyte ($\times 10^3/\mu L$) at day +100 | n=14 | 1015.00 [855.00–1370.00] | n=81 | 1970.00 [1490.00–2830.00] | <0.0001 | +0.748 |
| Platelet ($\times 10^3/\mu L$) at day +100 | n=14 | 221500.00 [181250.00–270250.00] | n=81 | 167000.00 [136000.00–212000.00] | 0.0069 | -0.455 |

Group comparisons were performed using the Mann–Whitney U test. The rank-biserial correlation coefficient (r) is reported as an effect size measure. Patients were stratified using the X-tile-derived PFS-optimized cut-off (31.53) for HALP at day +100. The high HALP group at day +100 was characterized by higher hemoglobin and lymphocyte counts and lower platelet counts compared to the low HALP group. HALP: Hemoglobin × Albumin × Lymphocyte / Platelet; IQR: Interquartile Range; PFS: Progression-Free Survival.

**Table S9.** Comparison of Hemoglobin, Albumin, Lymphocyte, and Platelet Levels Between Low and High HALP Groups — HALP at day +100, OS-optimized (cut-off 75.52).

| Biomarker | Low n | Low Median [IQR] | High n | High Median [IQR] | p | r |
|---|---|---|---|---|---|---|
| Hemoglobin (g/dL) at day +100 | n=68 | 12.15 [11.30–13.10] | n=27 | 12.30 [11.75–13.40] | 0.1907 | +0.173 |
| Albumin (g/dL) at day +100 | n=68 | 4.40 [4.20–4.70] | n=27 | 4.40 [4.10–4.60] | 0.6160 | -0.066 |
| Lymphocyte (×10³/μL) at day +100 | n=68 | 1500.00 [1207.50–1865.00] | n=27 | 2840.00 [2305.00–3405.00] | <0.0001 | +0.807 |
| Platelet (×10³/μL) at day +100 | n=68 | 190000.00 [151500.00–243750.00] | n=27 | 159000.00 [122000.00–181500.00] | 0.0009 | -0.440 |

Group comparisons were performed using the Mann–Whitney U test. The rank-biserial correlation coefficient (r) is reported as an effect size measure. Patients were stratified using the X-tile-derived OS-optimized cut-off (75.52) for HALP at day +100. The high HALP group at day +100 demonstrated significantly higher lymphocyte counts and lower platelet counts compared to the low HALP group. HALP: Hemoglobin × Albumin × Lymphocyte / Platelet; IQR: Interquartile Range; OS: Overall Survival.

**Table S10.** Comparison of Hemoglobin, Albumin, Lymphocyte, and Platelet Levels Between Low and High HALP Groups — ΔHALP, PFS-optimized (cut-off 25.91).

| Biomarker | Low n | Low Median [IQR] | High n | High Median [IQR] | p | r |
|---|---|---|---|---|---|---|
| Hemoglobin (g/dL) at day +100 | n=59 | 12.00 [11.25–13.00] | n=36 | 12.60 [12.03–13.35] | 0.0319 | +0.264 |
| Albumin (g/dL) at day +100 | n=59 | 4.40 [4.20–4.65] | n=36 | 4.45 [4.17–4.60] | 0.8232 | -0.028 |
| Lymphocyte (×10³/µL) at day +100 | n=59 | 1500.00 [1190.00–1900.00] | n=36 | 2595.00 [2037.50–3225.00] | <0.0001 | +0.643 |
| Platelet (×10³/µL) at day +100 | n=59 | 185000.00 [152500.00–235000.00] | n=36 | 162000.00 [122000.00–203500.00] | 0.0420 | -0.250 |

Group comparisons were performed using the Mann–Whitney U test. The rank-biserial correlation coefficient (r) is reported as an effect size measure. Patients were stratified using the X-tile-derived PFS-optimized cut-off (25.91) for ΔHALP (the change in HALP score between diagnosis and day +100). The high ΔHALP group, indicating greater recovery of immunonutritional indices following ASCT, was characterized by significantly higher hemoglobin and lymphocyte counts and lower platelet counts at day +100. ΔHALP: Change in HALP; HALP: Hemoglobin × Albumin × Lymphocyte / Platelet; IQR: Interquartile Range; PFS: Progression-Free Survival.

**Table S11.** Comparison of Hemoglobin, Albumin, Lymphocyte, and Platelet Levels Between Low and High HALP Groups — ΔHALP, OS-optimized (cut-off 42.10).

| Biomarkers | Low n | Low Median [IQR] | High n | High Median [IQR] | p | r |
|---|---|---|---|---|---|---|
| Hemoglobin (g/dL) at day +100 | n=70 | 12.10 [11.30–12.97] | n=25 | 12.60 [12.10–13.50] | 0.0560 | +0.259 |
| Albumin (g/dL) at day +100 | n=70 | 4.40 [4.22–4.67] | n=25 | 4.50 [4.10–4.60] | 0.5133 | -0.089 |
| Lymphocyte (×10³/µL) at day +100 | n=70 | 1515.00 [1212.50–2037.50] | n=25 | 2840.00 [2250.00–3300.00] | <0.0001 | +0.692 |
| Platelet (×10³/µL) at day +100 | n=70 | 187000.00 [153000.00–235000.00] | n=25 | 150000.00 [121000.00–182000.00] | 0.0037 | -0.393 |

Group comparisons were performed using the Mann–Whitney U test. The rank-biserial correlation coefficient (r) is reported as an effect size measure. Patients were stratified using the X-tile-derived OS-optimized cut-off (42.10) for ΔHALP. The high ΔHALP group demonstrated significantly higher lymphocyte counts and lower platelet counts at day +100. ΔHALP: Change in HALP; HALP: Hemoglobin × Albumin × Lymphocyte / Platelet; IQR: Interquartile Range; OS: Overall Survival.

**Table S12.** Diagnostic Performance of HALP Score Variables for Predicting Day +100 Treatment Response.

| **Variables** | **AUC** | **95% CI** | **CI Excludes 0.50** |
|---|---|---|---|
| HALP at diagnosis | 0.577 | 0.437–0.728 | No |
| HALP at day +100 | 0.517 | 0.355–0.690 | No |
| ΔHALP | 0.479 | 0.329–0.629 | No |

Receiver Operating Characteristic (ROC) analysis was performed to evaluate the diagnostic performance of HALP score variables for predicting day +100 treatment response, dichotomized as good response (sCR + VGPR; n = 79) versus other responses (PR + SD + PD; n = 16). The 95% confidence interval was estimated using non-parametric bootstrap resampling (1,000 replications). For all three HALP variables, the 95% CI included the null value of 0.50, indicating that the discriminative performance was not statistically distinguishable from chance. AUC: Area Under the Receiver Operating Characteristic Curve; CI: Confidence Interval; HALP: Hemoglobin × Albumin × Lymphocyte / Platelet; ΔHALP: Change in HALP; sCR: Stringent Complete Response; VGPR: Very Good Partial Response; PR: Partial Response; SD: Stable Disease; PD: Progressive Disease.

**Table S13.** Multivariate Cox regression with serum albumin in place of HALP (Model B alternative specification).

| Variables | Multivariate HR [95% CI] | p |
|---|---|---|
| **OS at diagnosis** | | |
| Albumin (g/dL) | 0.92 [0.45–1.89] | 0.813 |
| Age (years) | 1.00 [0.95–1.06] | 0.885 |
| Sex (male vs. female) | 0.40 [0.16–1.01] | 0.053 |
| ISS Stage (III vs. I–II) | 2.53 [0.76–8.44] | 0.131 |
| Maintenance therapy (yes vs. no) | 0.20 [0.07–0.54] | **0.002** |
| **PFS at diagnosis** | | |
| Albumin (g/dL) | 0.90 [0.55–1.45] | 0.653 |
| Age (years) | 0.99 [0.95–1.03] | 0.560 |
| Sex (male vs. female) | 0.39 [0.20–0.77] | **0.007** |
| ISS Stage (III vs. I–II) | 2.29 [0.94–5.61] | 0.070 |
| Maintenance therapy (yes vs. no) | 0.21 [0.09–0.47] | **<0.001** |
| **OS at day +100** | | |
| Albumin (g/dL) | 0.44 [0.13–1.49] | 0.189 |
| Age (years) | 1.00 [0.94–1.05] | 0.904 |
| Sex (male vs. female) | 0.40 [0.16–1.00] | 0.050 |
| ISS Stage (III vs. I–II) | 2.74 [0.89–8.43] | 0.079 |
| Maintenance therapy (yes vs. no) | 0.19 [0.07–0.51] | **0.001** |
| **PFS at day +100** | | |
| Albumin (g/dL) | 0.47 [0.19–1.18] | 0.108 |
| Age (years) | 0.99 [0.95–1.03] | 0.472 |
| Sex (male vs. female) | 0.36 [0.18–0.71] | **0.003** |
| ISS Stage (III vs. I–II) | 2.48 [1.08–5.69] | **0.032** |
| Maintenance therapy (yes vs. no) | 0.20 [0.09–0.44] | **<0.001** |

Multivariable Cox regression models with serum albumin substituted for HALP score, adjusting for age, sex, ISS stage (III vs. I–II), and maintenance therapy. Albumin was modeled as a continuous variable (per g/dL increase). The analysis was restricted to patients with known maintenance therapy status (n = 90). The proportional hazards assumption was verified using Schoenfeld residuals. Albumin alone did not demonstrate significant associations with OS or PFS in any model, whereas maintenance therapy was independently associated with both outcomes. HR: Hazard Ratio; CI: Confidence Interval; ISS: International Staging System; OS: Overall Survival; PFS: Progression-Free Survival. Bold p-values indicate statistical significance ($p \leq 0.05$).

## Supplementary Figures

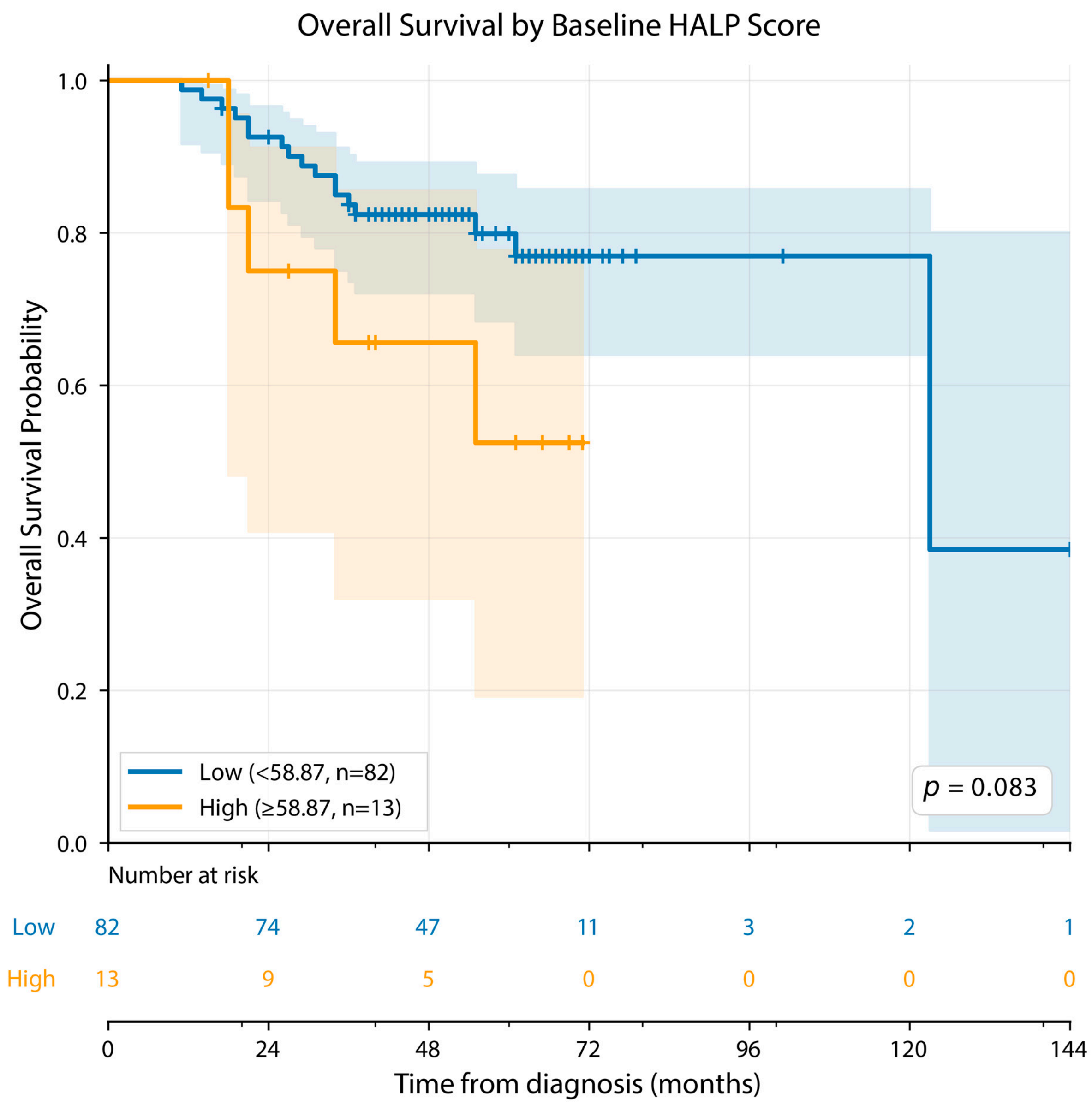


**Figure S1.** Kaplan–Meier analysis of overall survival (OS) according to baseline HALP score. The cut-off value was determined as 58.87 using the X-tile method. Patients were stratified into a low HALP group (<58.87; n = 82, blue) and a high HALP group (≥58.87; n = 13, orange). Censored observations are indicated by tick marks (+). Shaded areas represent 95% confidence intervals. Between-group comparison was performed using the log-rank test (p = 0.083). The number of patients at risk is shown below the curves at 24-month intervals. The log-rank test did not reach statistical significance.

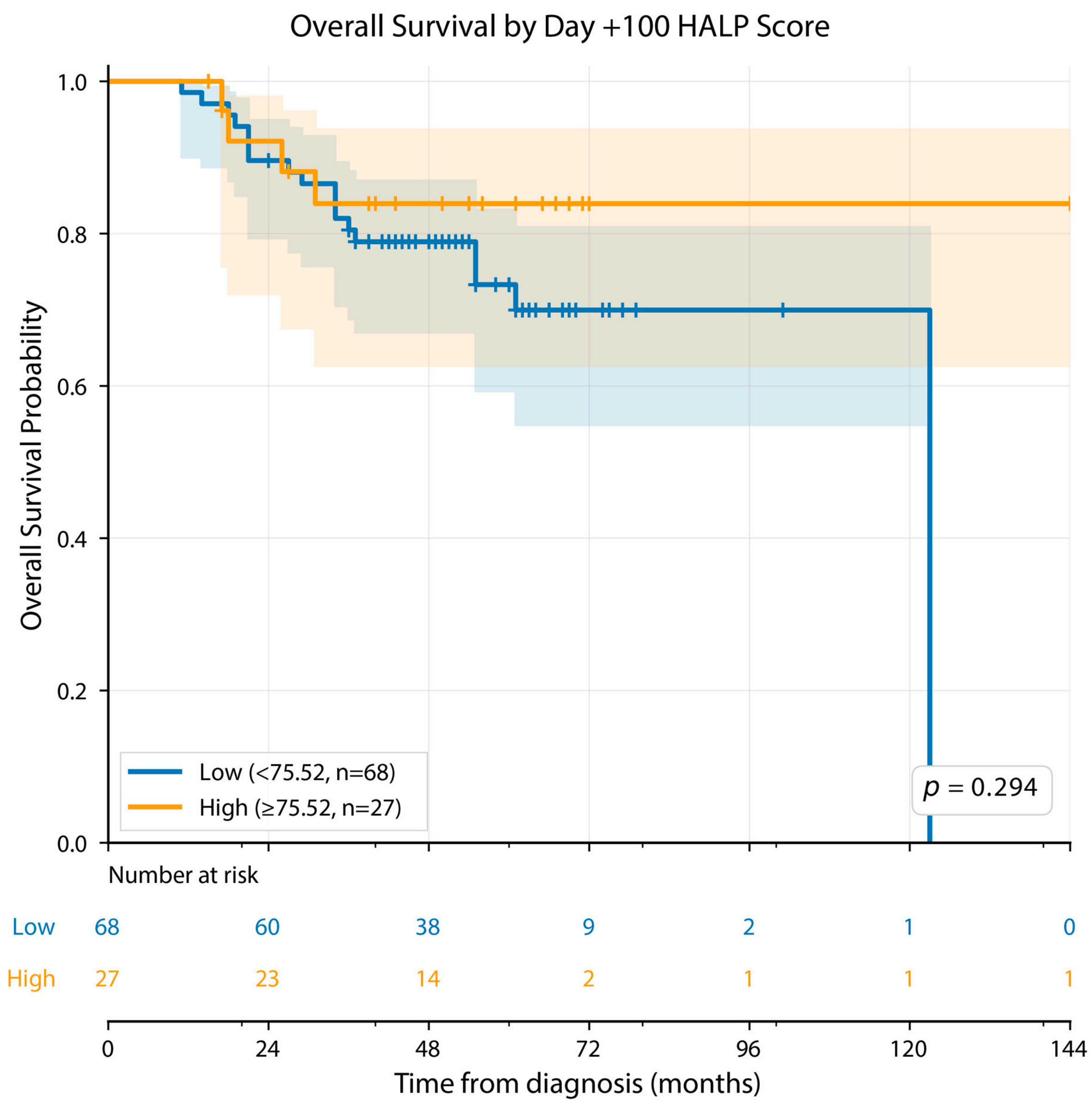


**Figure S2.** Kaplan–Meier analysis of overall survival (OS) according to HALP score at day +100 post-transplantation. The cut-off value was determined as 75.52 using the X-tile method, optimized for OS. Patients were stratified into a low HALP group (<75.52; n = 68, blue) and a high HALP group (≥75.52; n = 27, orange). Censored observations are indicated by tick marks (+). Shaded areas represent 95% confidence intervals. Between-group comparison was performed using the log-rank test (p = 0.294). The number of patients at risk is shown below the curves at 24-month intervals. No statistically significant separation between the groups was observed.

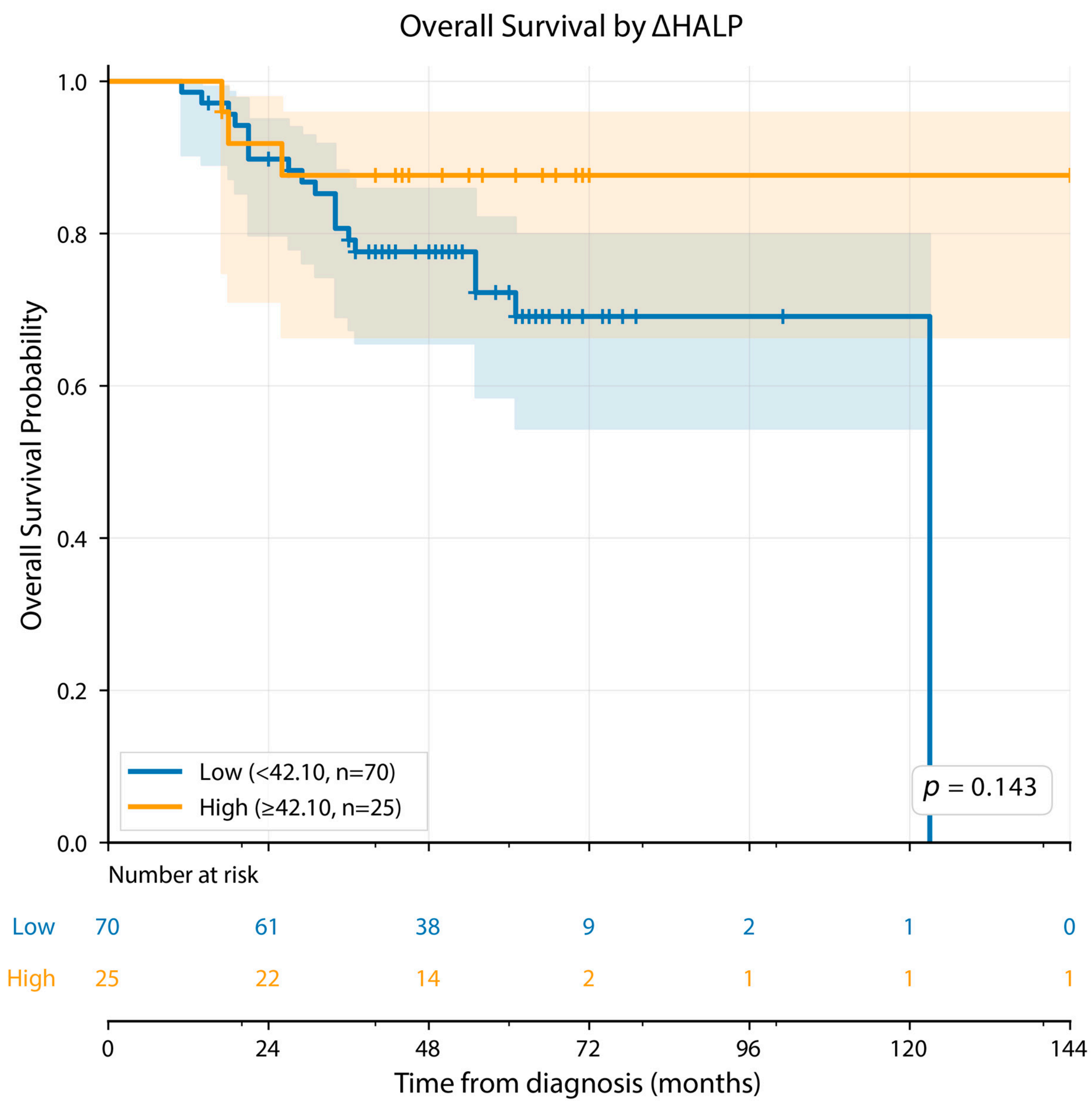


**Figure S3.** Kaplan–Meier analysis of overall survival (OS) according to ΔHALP. ΔHALP was defined as the difference between HALP at day +100 and baseline HALP. The cut-off value was determined as 42.10 using the X-tile method, optimized for OS. Patients were stratified into a low ΔHALP group (<42.10; n = 70, blue) and a high ΔHALP group (≥42.10; n = 25, orange). Censored observations are indicated by tick marks (+). Shaded areas represent 95% confidence intervals. Between-group comparison was performed using the log-rank test (p = 0.143). The number of patients at risk is shown below the curves at 24-month intervals. No statistically significant separation between the groups was observed.

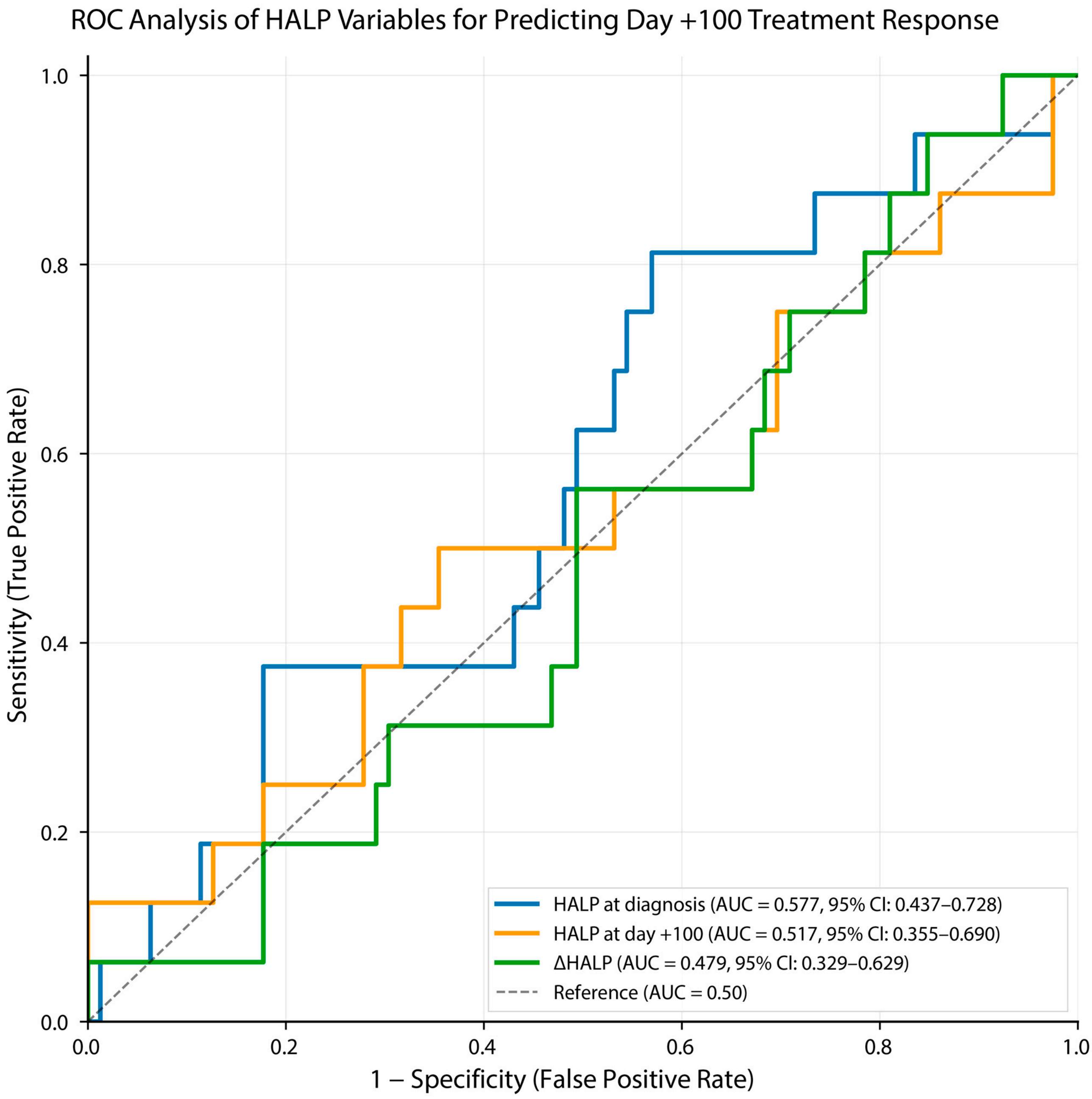


**Figure S4.** ROC curves are presented for HALP at diagnosis (blue; AUC = 0.577, 95% CI: 0.437–0.728), HALP at day +100 (orange; AUC = 0.517, 95% CI: 0.355–0.690), and ΔHALP (green; AUC = 0.479, 95% CI: 0.329–0.629). The dashed diagonal line represents the reference line of no discrimination (AUC = 0.50). The 95% confidence intervals were estimated using **non-parametric bootstrap resampling (1,000 replications)**.
